# Retinal vein occlusion in retinal racemose hemangioma: a case report and literature review of ocular complications in this rare retinal vascular disorder

**DOI:** 10.1186/1471-2415-14-101

**Published:** 2014-08-21

**Authors:** Xue-jiao Qin, Chao Huang, Kun Lai

**Affiliations:** 1 Department of Ophthalmology, Qilu Hospital of Shandong University, 107 Wenhua Xi Road, Box 639, Shandong PRC 250012, China

**Keywords:** Retinal racemose hemangioma, Retinal vein occlusion, Eye, Complication

## Abstract

**Backgroud:**

Retinal racemose hemangioma (RRH) is a rare congenital disorder that often co-occurs with other ocular complications. In this study, we present a case of RRH complicated with retinal vein obstruction in three branches and provide a review of ocular complications and associations with RRH.

**Case presentation:**

One case of RRH is presented. Fundus examination, fluorescein angiography (FFA) and optical coherence tomography (OCT) of the patient identified Group 3 RRH complicated with retinal vein occlusions in the superotemporal, inferotemporal, and inferonasal branches. Macular edema, which causes visual impairment, was detected. A brief literature review was also presented. The PubMed database was searched for RRH or related keywords to find reports of ocular complications or associations published on or before Dec. 31, 2013. A total of 140 papers describing167 RRH cases were found. The mean age of diagnosis was 22.97 years. Ocular complications were mentioned in 32 (19.16%) cases. Retinal vein occlusion (46.88%) was the major ocular complication in RRH, followed by hemorrhage (34.38%). Eight (4.79%) cases were associated with other ocular diseases such as Sturge–Weber syndrome , Morning glory disc anomaly and macroaneurysm.

**Conclusions:**

Although RRH is a relatively non-progressive condition, its complications may lead to vision loss and should be treated in time.

## Background

Retinal racemose hemangioma (RRH), also called retinal arteriovenous malformation [1,2] or retinal arteriovenous communication [3], is a congenital, non-hereditary, and sporadic phacomatosis that is characterized by the appearance of dilated and tortuous retinal vessels frequently extending unilaterally from the optic disc to the retinal periphery. Approximately 30% of RRH patients have coexisting arteriovenous malformations in the brain; this condition is known as Wyburn–Mason syndrome or Bonnet–Dechaumme–Blanc syndrome [4-6]. In rare cases, vascular malformations can also be found in the skin, kidneys, bones, and muscles of RRH patients.

RRH was once thought to be untreatable and does not cause hemorrhage. RRH alone may not cause any symptoms, and is thought to cause vision loss via various ocular complications. This paper presents a case of RRH with retinal vein occlusion (RVO) in three branches and provides a review of ocular complications or associations reported in papers published on or before Dec. 31, 2013.

## Case presentation

The current study complies with the protocols reviewed and approved by the independent ethics committee of Qilu Hospital of Shandong University and the tenets of the Declaration of Helsinki. Signed consent form was obtained from the patient.

**Figure 1:**
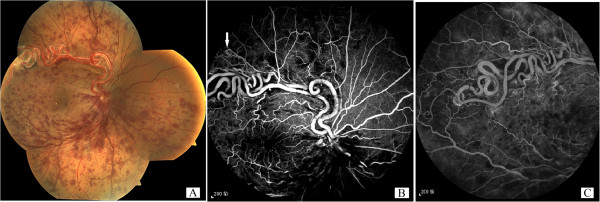
**Fundus and FFA of the diseased eye of the patient. A**: The fundus of the right eye showed a pair of enlarged and tortuous vessels extending from the optic disc. Hemorrhage was found in the temporal and inferior retina. **B**: FFA examination confirmed the presence of RRH and RVO in the superotemporal, inferotemporal, and inferonasal branches. **C**; The communication between the pair of tortuous vessels at the temporal retina.

**Figure 2:**
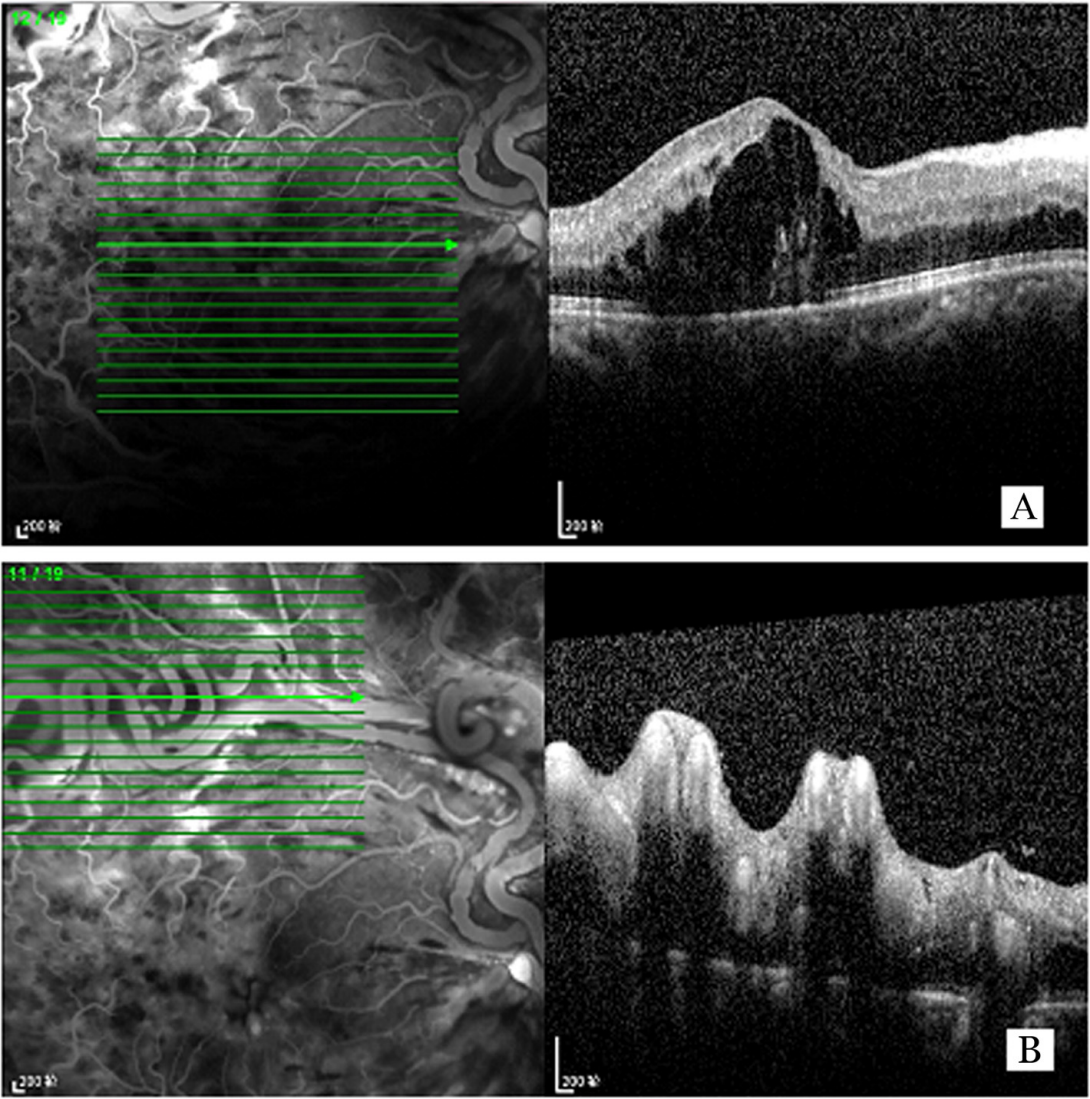
**OCT of the diseased eye of the patient. A**: cystoid macular edema was observed. **B**: the retinal surface was uneven in the area of distorted vessels.

A 58-year old male with sudden onset of blurred vision in his right eye for 10 days was examined in detail. The patient had a visual acuity of 20/100 in his right eye which was not correctable (mydriatic refraction +0.75 DS) at the time of presentation. Direct light reflex of the right pupil was weak. The left eye was refractive amblyopic with a best corrected visual acuity of 20/40 (+3.50 DS) without any other abnormalities. The patient was previously healthy and did not take any medication. The blood pressure was 128/88 mmHg, the blood sugar was 5.67 mmol/l, the blood lipid was slightly higher than normal (triglycerides 2.05 mmol/l, low density lipoprotein cholesterol 4.30 mmol/l). The fundus of the right eye showed a pair of enlarged and tortuous vessels extending from the optic disc. Flame-shaped hemorrhage was found in the temporal and inferior retina, along with dilated retinal veins, whereas macular central reflection was not identified (Figure 1A). FFA examination confirmed the presence of RRH and RVO in the superotemporal, inferotemporal, and inferonasal branches, indicating communication between the pair of tortuous vessels and hypofluorescence caused by hemorrhage and capillary nonperfusion (Figures 1B, 1C). The RRH was found to belong to Group 3 based on Archer’s classification because of the absence of capillary bed between the artery and the vein [3] and because of additional vessel anastomoses in the peripheral retina (Figure 1B, arrowed). OCT examination showed cystoid macular edema (Figure 2A) due to RVO. The retinal surface was uneven in the area of distorted vessels (Figure 2B). Magnetic resonance imaging (MRI) of the brain revealed no abnormality, and abdominal ultrasonography was normal despite the presence of a liver cyst.

### Literature review and discussion

Papers dated Dec. 31, 2013 or older were searched using “retinal arteriovenous communication,” “retinal arteriovenous malformations”, “Wyburn–Mason syndrome”, “retinal arteriovenous anastomoses”, “retinal racemose hemangioma”, and “retinal racemose angioma” as keywords in the PubMed database by two persons. The content of each search result was thoroughly checked to ensure its relevance to the topic of the study, especially for papers with ambiguous title. Non-English papers with expressions related to RRH in the English title, abstract, or keywords were included. Papers in Chinese were checked by reading the full text. Papers concerning complications in the *eye* of patients with RRH were recorded and analyzed.

PubMed search yielded 499 papers related to RRH. Except for reports on “Wyburn–Mason syndrome without retinal involvement”, 140 results (167 cases) were related to RRH, including so called “convoluted vessels”, “twin vessel”, “racemose aneurysm of retina”, and “retinal arteriovenous aneurysm”. Secondary arteriovenous communications were also excluded. Of the 167 cases mentioned in these papers, 152 reported the sex of the subjects (84, 55.26% females and 68, 44.74% males), 128 reported the age of diagnosis (range, 4 to 71 years; mean, 22.97 years), and 3 (1.79%) reported having bilateral occurrence of the disease [7,8]. Ocular complications or associations are summarized in Table 1. For patients with neovascular glaucoma secondary to RVO, only RVO was recorded. Ocular complications were reported in 32 (19.16%) cases, whereas ocular associations were reported in 8 (4.79%) cases.

**Table 1:** Ocular complications and associations of retinal racemose hemangioma

**Total RRH = 167 cases**	**Cases (percentage of total)**	**References**
**Complications**	**32 (19.16%)**	
Retinal vein occlusion	15 (46.88%)	
CRVO	8	[1,2,9-13]
CRVO	4	[7,12,14,15]
Hemi-central	1	[16]
Three Branches	2	[17], this report
Hemorrhage	11 (34.38%)	
Vitreous	5	[12,18,19]
Intra/sub-retinal	2	[12,20]
Macular	4	[7,18]
Rubeotic Glaucoma	2 (6.25%)	[12,21-23]
Macular edema	3 (9.38%)	[8,24,25]
Retinal detachment	1 (3.13%)	
Rhegmatogenous retinal detachment	1	[26]*
**Associations**	**8 (4.79%)**	
Morning glory disc anomaly	2 (25%)	[27,28]
Macular hole	1 (12.5%)	[29]
Sturge–Weber syndrome	2 (25%)	[30,31]
Vogt–Koyanagi–Harada syndrome	1(12.5%)	[32]
Duane type I retraction syndrome	1 (12.5%)	[33]
Macroaneurysm	1 (12.5%)	[34]

*It was hypothesized that vitreous hemorrhage over a long period may have preceded this.

Retinal vascular tumors are classified into four clinical categories, including retinal capillary hemangioma, retinal cavernous hemangioma, RRH, and retinal vasoproliferative tumor [35-37]. As a phacomatosis disorder, RRH can manifest at an early age. The average age determined by the current study is 22.97 years old, which is similar to the mean age of 23 years from 27 patients with Wyburn–Mason syndrome reported by Dayani and Sadun [38]. The average age determined by the current study in patients with RRH is significantly younger than the average age of 42 years from 13 RRH patients in the study by Mansour et al. (7), who also reported the disease as “arteriovenous anastomoses of the retina”. Retinal vascular abnormality usually exhibits no symptoms, and is not easily determined compared with malformations in other body parts. Patel (40) reported a case of Wyburn–Mason syndrome with vascular abnormalities in the face, orbit, and brain (but not in the retina) of a newborn. RRH was once thought to be non-progressive, and patients can often continue to have good vision [39,40]. The longest follow-up period was 27 years without any progression in the retinal or cephalic condition [40]. A case of self-regression was also reported [41]. However, previous reports indicated that vessel dilation and elongation would occur over time in previously normal vessels [42] and that visual impairment would occur because of late ocular complications, particularly ischemic complications [14,17,21]. The current study found that RVO is the most common complication of RRH. RVO accounted for 45.46% of the total number of complication cases, while hemorrhage only accounted for 33.33%. Venous occlusion in RRH was attributed mainly to abnormal turbulent blood flow in the veins. In patients with RRH, the veins that connect directly to the arteries are subjected to arterial blood pressure. High blood pressure causes irregular venous wall thickening, endothelial damage and proliferation, and thrombosis [43]. In some cases, severe thrombosis and further fibrosis can cause the malformed vessels to close spontaneously [44-46]. In the present study, the superotemporal, inferotemporal, and inferonasal venous branches were found to converge into a single trunk while the superonasal venous branch drained to another trunk in the disc. This phenomenon is called incomplete central retinal vein occlusion. The elevated level of blood lipid in this patient may also have induced the development of RVO. RVO is also considered a complication of cerebral arteriovenous malformation [47]. RVO and other related macular edema or secondary neovascular glaucoma are all vision-threatening conditions. Although racemose hemangioma is not easily treated, its complications should be handled accordingly to retard vision deterioration. Procedures for slowing vision deterioration include laser therapy for RVO, retrobulbar or intraocular injection of triamcinolone for macular edema, vitrectomy for vitreous hemorrhage, and drainage valve implantation for glaucoma. New anti-vascular endothelial growth factor agents have been tested for treatment of malformed vessels or macular edema complicated with RVO [24,48-50]. However, the association of RRH with other congenital vascular malformations, such as Sturge–Weber syndrome and macroaneurysm, continues to make it a challenging condition for doctors.

## Conclusion

As a congenital disorder, RRH may complicate or associate with various ocular conditions; clinicians should pay attention to these conditions and take action to preserve vision.

## Consent

Written informed consent was obtained from the patient for publication of this case report and any accompanying images. A copy of the written consent is available for review by the Editor of this journal.

## Abbreviations

(RRH): Retinal racemose hemangioma; (RVO): Retinal vein occlusion; (FFA): Fluorescein angiography; (OCT): Optical coherence tomography; (MRI): Magnetic resonance imaging.

## Competing interests

The authors declare that they have no competing interest.

## Authors’ contributions

XJQ conceived the study; XJQ and CH conducted the clinical examinations; XJQ and KL conducted the PubMed search, data analysis, and data interpretation; XJQ, CH, and KL wrote and revised the manuscript. All authors read and approved the final manuscript.

## Pre-publication history

The pre-publication history for this paper can be accessed here:

http://www.biomedcentral.com/1471-2415/14/101/prepub
